# 

**Published:** 1856-11

**Authors:** 


### CONTENTS.

ORIGINAL COMMUNICATIONS— PAGE.

Albumanuria. By F. B. Norcum, M.D. .......... 487
A Case of Complicated Labor. By Hiram Nance, M.D. Lafayette, Ill... 493
Report on the Meteorological Characteristics, and more Important Diseases Prevalent in Chicago, during the Summer of 1856. By N. S. Davis, M.D. .......... 497

SELECTIONS, ..........

New York Medico-Chirurgical Society—Tetanus, &c.. .......... 509
Sulphate of Cinchona equal to Sulphate of Quinia, in the Treatment of Intermittents. By L. Faulkner, M.D. Halifax County, Va. .......... 514
Crystalized Sub-Acetate of Lead, and Tincture of Digitalis in Hypertrophy of the Heart. Reported by W. F. Jones, M.D. Petersburg, Va. .......... 517
Diagnosis of Injuries of the Shoulder-Joint, .......... 519
Extract from a Notice of Recent Researches on the Origin of Entozoa, more especially of Tape-Worms. By Allan Thompson, M.D. F.R.S. London and Edinburgh, and Professor of Anatomy in the University of Glasgow, .......... 521

BOOK NOTICES, ..........

Bennet on Uterine Pathology—Gardner on Sterility, .......... 524
Medical Jurisprudence. By Alfred S. Taylor, M.D. F.R.S. .......... 525
The Dissector's Manual of Practical and Surgical Anatomy. By Erasmus Wilson, F.R.S. &c. .......... 525
A Treatise on the Practice of Surgery. By Henry H. Smith, M.D. ....... 525

EDITORIAL, ..........

A Parting Word, .......... 528
Prize Essay, .......... 529
Ununited Fractures, .......... 530

MISCELLANEOUS ITEMS, ..........

Legal Responsibilities—Extensive Injury During Pregnancy, .......... 531
Sun's Rays in Consumption—Death from Drinking Naphtha, .......... 632
Benzoin in the Treatment of Chronic Dysentery—Professional Reputation, .......... 533
Works Published by the Sydenham Society, .......... 534

